# Thymus-Pineal Gland Axis: Revisiting Its Role in Human Life and Ageing

**DOI:** 10.3390/ijms21228806

**Published:** 2020-11-20

**Authors:** Rita Rezzani, Caterina Franco, Rüdiger Hardeland, Luigi Fabrizio Rodella

**Affiliations:** 1 Anatomy and Physiopathology Division, Department of Clinical and Experimental Sciences, University of Brescia, 25123 Brescia, Italy; c.franco@studenti.unibs.it (C.F.); luigi.rodella@unibs.it (L.F.R.); 2 Interdipartimental University Center of Research “Adaption and Regeneration of Tissues and Organs-(ARTO)”, University of Brescia, 25123 Brescia, Italy; 3 Johann Friedrich Blumenbach Institute of Zoology and Anthropology, University of Göttingen, Lower Saxony, D-37073 Göttingen, Germany; rhardel@gwdg.de

**Keywords:** thymus-pineal axis, thymic and pineal factors, melatonin, ageing, rejuvenation

## Abstract

For years the thymus gland (TG) and the pineal gland (PG) have been subject of increasingly in-depth studies, but only recently a link that can associate the activities of the two organs has been identified. Considering, on the one hand, the well-known immune activity of thymus and, on the other, the increasingly emerging immunological roles of circadian oscillators and the rhythmically secreted main pineal product, melatonin, many studies aimed to analyse the possible existence of an interaction between these two systems. Moreover, data confirmed that the immune system is functionally associated with the nervous and endocrine systems determining an integrated dynamic network. In addition, recent researches showed a similar, characteristic involution process both in TG and PG. Since the second half of the 20th century, evidence led to the definition of an effectively interacting thymus-pineal axis (TG-PG axis), but much has to be done. In this sense, the aim of this review is to summarize what is actually known about this topic, focusing on the impact of the TG-PG axis on human life and ageing. We would like to give more emphasis to the implications of this dynamical interaction in a possible therapeutic strategy for human health. Moreover, we focused on all the products of TG and PG in order to collect what is known about the role of peptides other than melatonin. The results available today are often unclear and not linear. These peptides have not been well studied and defined over the years. In this review we hope to awake the interest of the scientific community in them and in their future pharmacological applications.

## 1. Introduction

Organisms use both circadian and circannual rhythms for time measurements. The rhythms are linked to fluctuating factors such as light and temperature [1,2,3]. Light strongly affects behavioural and metabolic rhythms along with a host of numerous processes that determine the temporally correct expression of genes [4]. Since long, the evolutionary “invention” of circadian rhythms has been interpreted as a consequence of avoiding damaging radiation [5,6,7]. Importantly, this includes oxidative stress resulting thereof [8,9]. Meanwhile, it has been shown that many forms of stress can affect circadian oscillators in extant organisms [10,11]. A mechanism by which oxidative stress can affect a circadian oscillator has been described: reactive oxygen species (ROS) decrease the concentration of nicotinamide adenine dinucleotide (NAD^+^), i.e., the substrate and activator of sirtuins, among which situin 1 (SIRT1) acts as an amplitude-enhancing accessory component of oscillators [4]. Interestingly, multiple interactions between the circadian system, oxidative stress, immune system and melatonin (MT) have become apparent [12]. These nexuses are also evident at the level of gene expression and comprise overlapping epigenetic mechanisms of chromatin remodelling [13]. The aspect of overlapping regulation by a multi-functional network has recently received support by the identification of numerous microRNAs (miRNAs) and miRNA-sponging circular RNAs (circRNAs) that are involved in pro- or anti-inflammatory responses and in the control of core and accessory oscillator components including SIRT1. Conversely, numerous of these immunologically relevant non-coding RNAs are controlled by circadian output factors such as D-box-binding protein (DBP), by SIRT1 and MT, as well [14].

The rhythms are used by organisms to verify day/night length and to control physiological processes. A well-functioning circadian system that operates at sufficiently high amplitudes and is devoid of misalignment and internal desynchronization has been judged to be of utmost importance for the maintenance of human health [15,16]. Disruption of the circadian clock contributes to numerous pathologies, including cardiovascular diseases, cancer, neuroendocrine disorders, mood disorders, type 2 diabetes and metabolic syndrome [4,17,18,19]. Moreover, during ageing, the dyscoordination of rhythms and dysfunctions of many functions of the organism, such as neuroendocrine and immune systems induce substantial problems in elderly people [20].

A growing body of data suggests that the immune system is functionally associated with the nervous and endocrine systems, thereby forming an integrated, balanced network [21]. In addition, age-associated alterations in the neuroendocrine systems are believed to be responsible of the development of age-associated diseases [22,23]. In this regard, the co-involution of the thymus gland (TG) and of the pineal gland (PG) could play an important role in ageing and the “time of death”, even though other factors can additionally induce functional deficits in these organs and the entire body [24,25].

In particular, considering the central immunological role of the TG, its decline in the course of immunosenescence and the resulting necessities of immune remodelling are in accordance with the conclusion that the functionality of the immune system is the strongest predictor of human longevity and healthy aging [26,27].

At the same time, the removal of PG in mice induces a decrease in weight, a complete destruction of the TG and the impairment of the immune system accompanied by wasting diseases [25,28,29]: these information confirm that the involution of the TG and PG goes hand in hand because they mutually influence each other in the context of immune system regulation and they act as a functional unit, known as the thymus-pineal axis (TG-PG axis) [30,31].

The TG-PG axis was identified in the second half of the 20th century when many results suggested an overlap between the functions of these glands [23]. The important thrust for this hypothesis was keyed to the notion that these glands act *in tandem* for regulating cells, tissues and body growth [32].

This review aims to summarize and underline the available data about the TG-PG axis in order to suggest that these glands have many and often different molecular functions, but they also interact in manifold ways via highly complex networks during life and ageing. Moreover, in spite of the results reported here on changes of these glands, we do not suggest or intend to demonstrate an exclusive role of the TG-PG axis in youth and old age. Instead, we would like to stimulate and request attention for preparing adequate therapeutic strategies that will be important for the improvement of age-related diseases. Moreover, we focus the scientific attention on all the products of TG and PG in order to collect what is known about the role of peptides other than melatonin. The results available today are often unclear and ambiguous. These peptides have not been studied very much over the years, so we hope that making them one of the main topics of this review may awaken the interest of the scientific community in them and in their future pharmacological applications. In doing so, the first part will consider the morphological analysis of these two glands from beginning of life to ageing. Next, the concept of their secretion and their relationship with ageing will be discussed. Finally, a very important point will be to elaborate the networks which these glands have during life and to underline the most important details.

## 2. Revisiting the Thymus Gland

Traditionally, TG is composed of a cortex and a medulla which contain developing thymocytes in the network of epithelial and stromal cells [33]. Its cytoarchitecture is evolutionarily preserved in different vertebral species and it has the same immunological functions [34,35]. Moreover, an impressive paradoxical feature of this organ is that it is affected by age-associated changes known as immunosenescence [36,37]. Undeniably, the immunosenescence involves many intrinsic and extrinsic factors not only linked to TG, but it is also associated with changes in other organs such as PG [38].

**Figure 1:**
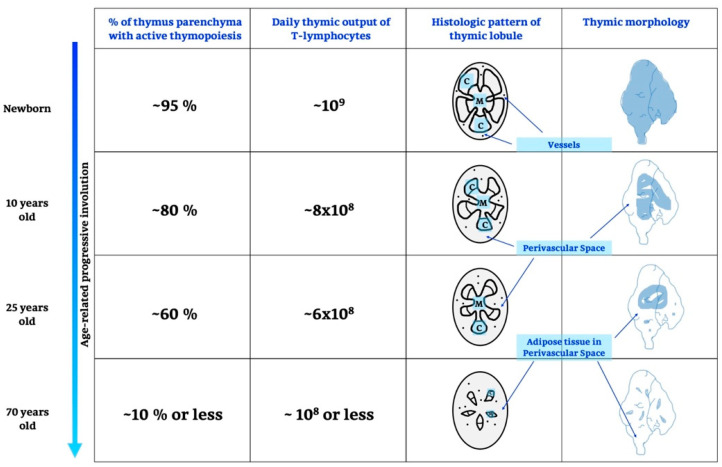
Age-related thymic involution. During growing and ageing thymus undergoes different changes, in particular, there are variations on thymic functionality, expressed as% of active parenchyma and number of T-lymphocytes produced, moreover, also a histological, morphological and anatomical remodelling is recognized. C, cortex; M, medulla.

It is known that the TG develops in fetal life, peaking prior to puberty with a progressive decline into adult life; after adolescence, it shrinks and generates few and fewer T cells [39]. Details are shown in Figure 1 and below.

### 2.1. Life and Involution of the Thymus Gland

The time when the epithelial anlage of TG is developing and when it becomes responsive to immigrating lymphoid progenitor cells varies from species to species [39]; in particular, TG is colonized on the third day after fertilization in zebrafish [40], on embryonic day 12 in mice [34] and on embryonic day 90 in humans [41]. The important step of thymic development is a rapid growth with a strong proliferation and differentiation of epithelial and lymphoid cells [42]. During life, the proliferation and enhanced activity of some autoreactive cells, which gradually wear down cells and intercellular materials, contribute to and may even induce ageing. The enhanced and mass function of autoreactive cells leads to autoimmune diseases and natural death. This means that the involution of TG is not a part of the organism’s involution, because it is considered a very important lifespan-pacemaker [25]. In humans, thymic involution begins around the time of birth and continues throughout life [43]; in mice, TG reaches its maximum size at 4 weeks of age, and successively it begins to shrink [44,45].

Factors outside the immune system seem to have many effects on TG functions; among these factors, one would consider hormones such as estrogens, androgens, glucocorticoids, progesterone, and somatostatin [46]. In particular, some studies demonstrated that estrogens but not progesterone block T cell development in the TG [47]; other data underlined that androgens cause the destruction of thymic cells by stimulating the secretion of many epithelial cells-derived factors that, in turn, induce involution of this organ [48]. Interestingly, other researchers suggested that progesterone and its receptors, present on lymphoid cells, cause pregnancy-induced thymic involution by inducing a loss of T cell precursors [49]; moreover, these authors showed that loss of thymic involution during pregnancy results, instead, in decreased fertility and increased fetal loss. Thus, these considerations indicate that the involution is no good in terms of ageing, but, with regard to reproduction, is important for maintaining the hope of pregnancy.

Concerning the mechanisms of TG involution, a number of hypotheses have been suggested, which are, sometimes, conflicting. Some researchers have reported a correlation between thymic involution and sex hormones, while others have found the opposite and also arrived at the conclusion that the effects of hormones on TG have no important role in its involution. As far as a role of sex hormones is assumed, thymic shrink might be associated with their increased concentrations at puberty. Correspondingly, exogenously administered androgen or estrogen inhibit thymopoiesis, whereas gonadectomy increases TG size. This link between sex steroids and thymic involution had been initially suggested over 100 years ago by experimental studies showing that castration resulted in increased TG size [50,51].

Moreover, the last and more recent hypothesis relegates the causes of thymic shrink to intrinsic haemopoietic defects, which are apparent in a reduced proliferative capacity of early T lineage progenitor (ETP) cells [52]. ETP cells represent the earliest intrathymic precursors and are present in the developed thymus within the so-called TN1 compartment. This compartment increases by age, but is heterogeneous and contains many non-ETP cells. Thus, the composition of the TN1 compartment changes by age and the decline of ETP cells may be interpreted in terms of an alteration of the intrathymic microenvironment. In this context, it might also be perceived that several immune relevant factors, among them leukemia inhibitory factor (LIF) and oncostatin M (OSM), both belonging to the IL-6 cytokine family, increase with age and seem to change the intrathymic microarchitecture leading to thymic atrophy [53]. However, the role of OSM requires further clarification, since a more recent study showed that OSM-deficient mice exhibited thymic atrophy, changed thymic microarchitecture and increased numbers of apoptotic thymocytes [54].

Overall, in the current state of our knowledge, it seems important to consider multiple reasons for the mechanisms of both thymopoiesis and thymic involution. Four points requiring additional research are of particular interest.

The first point is related to the demonstration that TG, after castration, increases in size, but a convincing mechanistic nexus has not been demonstrated. It would be necessary, therefore, to identify a direct connection with the hypothalamic-pituitary axis. The hypothalamic gonadotropin releasing hormone (GnRH) stimulates the adenopituitary gland to produce luteinizing hormone (LH) and follicle stimulating hormone (FSH) that, in turn, induce gonadal secretion of sex steroids. A negative feedback by elevated androgen concentration inhibits the hypothalamic GnRH production. In the female, the corresponding feedback circuit is more complex because estrogens have both positive and negative effects on GnRH formation [51,55].

**Figure 2:**
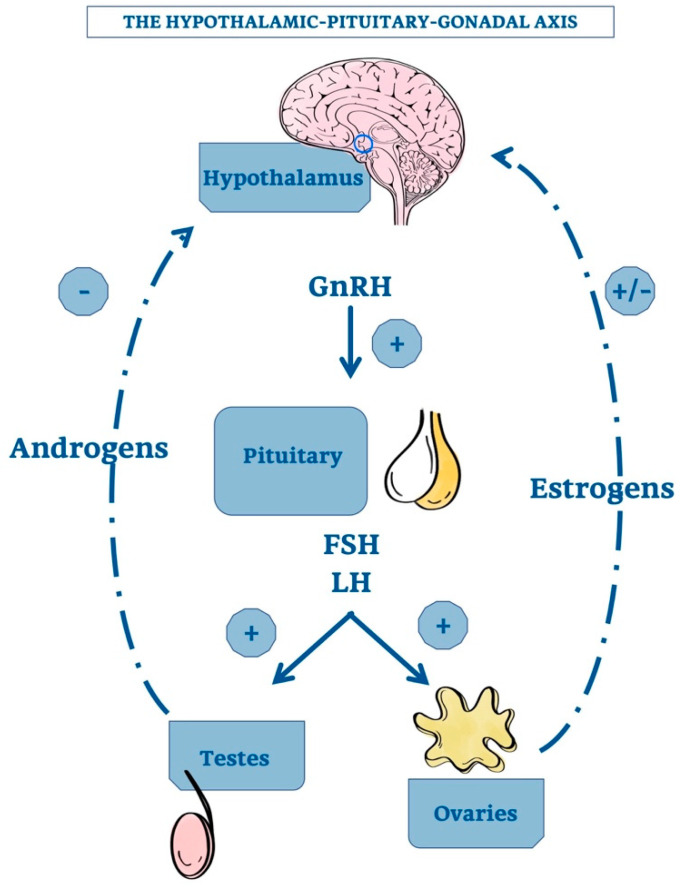
The hypothalamic-pituitary-gonadal axis. Schematic representation of the relationship among the hypotalamus, the pituitary gland and the gonadals. The hypotalamic GnRH positively regulate pituitary’s activity. In addition, also androgens and estrogens exert a variable feedback directly on hypotalamus, allowing its correct functioning. +, stimulatory effect; -, inhibitory effect; GnRH, Gonadotropin-releasing Hormone; FSH, Follicle-stimulating Hormone; LH, Luteinizing Hormone.

Figure 2 shows the hypothalamic-pituitary-gonadal axis with the positive and negative effects of androgens and estrogens on GnRH production.

Of note, GnRH is also produced locally in TG [56]. However, a mainly autocrine or paracrine role of thymic GnRH may be assumed, since its expression is strongly correlated with that of its receptor in the thymus [56]. GnRH amounts and concentration increase after castration and this effect is reversed by testosterone. In addition, alterations of both TG volume and proGnRH processing contribute to these changes. Therefore, according to Montecino-Rodriquez and colleagues, the increased number of thymocytes in gonadectomized mice seems to be due to the stimulation of GnRH production and/or absence of sex steroids [51].

The second point is that a sex dimorphism is evident following gonadectomy because the increase in size is more prominent in males relative to females [57]. On the basis of these data, it is possible to suggest that sex steroids are important effectors of thymic involution, but they are dependent on gender. Whether sex steroids are decisive factors that are responsible for thymic shrinkage is testable, since, in this case, their removal should prevent thymic involution. However, according to Montecino-Rodriquez et al., this is not the case [51]. Two reports have concluded that the effects of gonadectomy on increasing TG weight or cellularity is transient. Animals that were ovariectomized at 2 months of age exhibited thymic involution at 20 months of age [57,58]. We agree with the interpretation by Montecino-Rodriquez et al., who indicated that the organisms can adapt to the new situations, such as gonadectomy, destruction of physiological endocrine circuits and, thereby, restore the normal TG size and its fate to shrink [51]. Thus, this concept needs to be considered and evaluated for therapeutic strategies.

The third point is that the increase in size after gonadectomy is linked to age because it is lower in old respect to young rodents as demonstrated by many investigators [58]. The increase in TG size was 80% and 40% in castrated male mice at 3- or 18-months of age, respectively, relative to age-matched sham-operated controls. Instead, age-related declines in growth hormone (GH) and insulin-like growth factor-1 (IGF-1) production have been suggested to contribute to thymic involution, since TG size increases after their administration [59,60].

The last and more recent hypothesis is linked to a revised model in which thymic shrinkage results from age-related intrinsic and extrinsic haemopoietic factors, which affect the development and functions of T-cell precursors [52], as outlined in a preceding paragraph. It will be a matter of future research to identify the factors and processes that lead to causative changes in the thymic microenvironment, in cell composition, in the ETP/non-ETP balance, and the reduction of ETP proliferative capacity. A compromised microenvironment may lead to a vicious cycle in which the deteriorating processes further decrease the functionality of lymphoid cells and, thus, promote the progressive involution of the organ. As generally in aging tissues and particularly in the context of immunosenescence, the question will also be in TG that of the contributions of genetically damaged cells that do not undergo apoptosis, but survive under conditions of a senescence-associated phenotype (SASP), release pro-inflammatory cytokines and other factors that contribute to inflammaging [61,62]. Functional losses can also be caused by garb-aging, along with decreases in mito-/autophagic capacity [62], i.e., deteriorations that concern many cell types beyond ETPs and differentiated thymocytes. If these processes turn out to be of relevance to the TG, the remaining problem will be that of why this organ’s decline is apparently more rapid than that of other tissues. Perhaps, the systemic view including the TG-PG axis and further nexus within an even larger network may provide new insights.

### 2.2. Thymic Factors and Their Effects

Nowadays, TG is considered an organ performing two functions together; it has a central role in the lymphoid system, but it is also regarded as an important organ of the endocrine system. Of note, some thymic endogenous factors are produced without having a primary immune function [25].

Many endogenous factors produced by the TG have been identified, but the most important among them are *thymulin*, *thymosin*, *thymopoietin* (also called *thymin*), and *thymic humoral factor* [63]. These hormones are produced by epithelial cells, which also synthetize cytokines, such as IL-1 and IL-6, granulocyte-macrophage colony stimulating factor and granulocyte colony stimulating factor, which play an important role in thymocyte differentiation.

Moreover, many investigations have underlined that thymic hormones act on the PG, modulating the synthesis of MT [64,65]. In addition, the human and rat TGs express the biosynthetic enzymes of MT formation, i.e., of a molecule that has, in addition to its role as a hormone, intracrine, autocrine and paracrine functions [65,66,67]. The synthesis of MT in TG is no longer surprising, not only because its formation has been meanwhile demonstrated in numerous extrapineal tissues [68], but also keeping in mind that the overall amount of extrapineally formed MT exceeds that in PG and circulation by orders of magnitude [69]. Moreover, the increasing number of cases of preferentially mitochondrial MT biosynthesis has led to the assumption that it may be produced in many, perhaps the majority of vertebrate cells [70]. Therefore, the decisive point is not that of whether an organ such as the TG is capable of synthesizing MT, but rather that of the amounts that are released, either to the circulation or towards infiltrating cells, in other words, whether it contributes to intra-organismic communication. With regard to TG, this aspect deserves further clarification, especially as the organs with highest MT synthesizing capacity, such as the gastro-intestinal tract, release MT at substantial rates only under exceptional conditions [71].

#### 2.2.1. Thymulin

Thymulin (formerly called “facteur thymique sérique” or FTS) is a metallopeptidic hormone playing a role in intrathymic and extrathymic T cells [72,73]. It consists of a nonapeptide, to which a zinc ion is bound (at Asn, Ser4 and Ser8), which is required for its biological activity [74]. The use of the term FTS has been somewhat inconsistent: sometimes this has been used as a synonym of thymulin, sometimes it only refers to the peptide, or it is used when antibodies do not safely distinguish between the zinc-containing complex and the peptide devoid of zinc.

Anyway, the thymulin production is regulated both in vivo and in vitro by a feedback mechanism [75,76]. In humans, FTS is synthesized by the thymic reticulo-epithelial tissue (RE), which progressively undergoes an involution. This determines an age-related decline in serum FTS levels that begins around 20 years of age resulting in a complete absence of this factor in blood between the 5th and 6th decade of life [77].

In addition, some evidence suggested a hypophysiotropic activity of thymulin by stimulating LH and adrenocorticotropic hormone (ACTH) secretion by the pituitary gland. The secretion of ACTH is mediated by cAMP, whereas increases in cGMP are only observed at high agonist concentrations and may be physiologically irrelevant [78].

**Figure 3:**
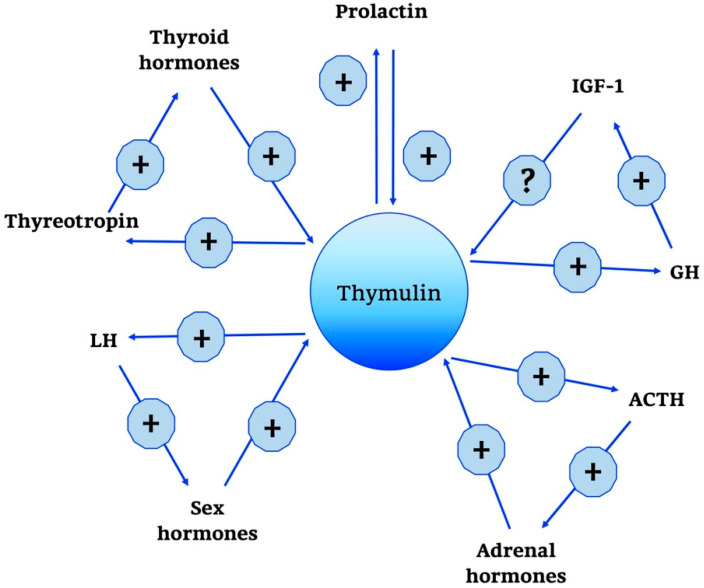
Thymulin neuro-endocrine functions. Thymulin neuro-endocrine functions are summarized in the scheme. +, stimulatory effect; -, inhibitory effect; ?, no data; GH, Growth Hormone; IGF-1, Insulin Growth Factor-1; ACTH, AdenoCorticoTropic Hormone; LH, Luteinizing Hormone.

Moreover, it has been known that thymulin has positive and negative effects on several hormones, as summarized in Figure 3, such as GH, thyroid-stimulating hormone (TSH), and gonadotropin in incubated rat pituitary fragments [79,80]. Other studies have confirmed the existence of a feedback action of thymulin on its own secretion.

Interestingly, ACTH is not only controlled in its secretion by thymulin, but also stimulates thymulin release by thymus fragments in culture [81,82]. Other regulatory influences on thymulin release have been described for prolactin, GH and thyroid hormones [83].

In addition, regarding the CNS-related and other non-immunological effects, it should be noticed that thymulin formation has been also found in astrocytes and in keratinocytes of the skin [82]. Importantly, the influence of thymulin on the pituitary gland declines with the age and this may imply that ageing desensitizes the pituitary gland to thymic signals [76].

More recent evidence underlined the important role of thymulin as a signalling molecule for interactions between immune, endocrine, and nervous systems that concern anti-inflammatory, anti-oxidant and anti-hyperalgesic functions [76,82,84]. In the last years, researchers focused on the potential therapeutic role of thymulin as an anti-inflammatory agent; in particular, it has been emphasized that, among all the thymic peptides that could have various impacts on mature immune cells and central endocrine systems, thymulin is the one that exerts mostly inhibitory effects on immune responses [85].

Lunin and colleagues considered this when analyzing its possible suitability in controlling severe experimental autoimmune encephalomyelitis (sEAE) [85]. Previous studies had demonstrated additional beneficial actions of this molecule, such as its ability in preventing LPS-induced pancreatic cells damage and attenuation of experimental allergic asthma [85,86,87]. In mechanistic terms, the suppression of NF-κB signalling was demonstrated in LPS-stimulated lymphocytes, since similar effects were also obtained by inhibitors of the NF-κB pathway [85,88,89]. Later, other studies considered effects of thymulin on another inflammatory pathway, the SAPK/JNK signalling cascade that is normally stimulated by stress signals such as UV radiation, temperature changes and oxidative damage. Data from Lunin and colleagues, in fact, reported that thymulin could also intervene in this pathway, having an overall synergic effect with IKK inhibitor XII [85,90]. Collectively, actually available results show that thymulin acts by modifying the differential and selective release of pro-inflammatory cytokines. Findings are encouraging for exploring potential future therapeutic applications of this molecule in several areas, e.g., as an immuno-modulatory factor for the paediatric treatment of respiratory distresses [89].

#### 2.2.2. Thymosin

The first references to thymosin date back to 1966, when, in an attempt to better define the endocrine activity of the TG, Goldstein et al. identified a product which stimulates the incorporation of ^3^H-thymidine into mesenteric lymph node cells [91]. This molecule was named thymosin, but its identification was only the first step in the discovery of a complex world of multiple thymosins. 41 years later, Goldstein himself, author of the first publication, explained that the molecule, initially denominated as thymosin, corresponded to what is now known as thymosin fraction-3 (TF3) [92].

In our actual understanding, thymosins represent a series of immunoactive polypeptides. Progress in purification procedures allowed to discriminate numerous functionally different peptides, part of which belong to the more generally known thymosin fractions-3 and -5 [92]. According to isoelectric focusing patterns, three groups were distinguished, α-, β-, and γ-thymosins, with IEPs respectively, below pH 5, between pH 5 and 7, and above pH 7, which remained to be highly heterogeneous [93]. Thymosin fraction-5 (TF5) alone contains over 40 different molecules [94]. However, many components of these fractions are structurally and physiologically unrelated. Some of them and also some of their precursors (prothymosins) are transported into and acting within the nucleus, e.g., as histone H1-chaperones [95] and, perhaps, as transcription factors or their modulators [96,97]. Thymosins β4 (Tβ4) and β10 (Tβ10) are actin monomer-binding peptides [98], others initiate signalling pathways by interacting with membranes, such as in the case of thymosin α1 [99], whereas further thymosins are known to be immune modulators.

However, many of them are not exclusively produced in the thymus, but are widely expressed, such as Tβ4, which is formed in all nucleate cells of the body. Thymosin β1 has turned out to be a slightly truncated ubiquitin variant [100]. On the contrary, others may be more thymus-specific.

The multiplicity of factors that are widely non-homologous, their localization in different cell compartments and their expression in many organs are certainly substantial obstacles for obtaining a coherent picture of the thymosins. In the following, we shall, therefore, focus on the immunological actions of these factors, keeping in mind that important members of them are not only expressed in the thymus, but also elsewhere, often ubiquitously, and that their presence in the circulation is not *per se* an indicator of thymic origin.

Despite the heterogeneity of fractions, experiments and clinical applications were often conducted on the basis of these mixtures rather than single purified compounds. For example, TF5 was the first thymosin preparation in clinical use [101].

Investigations demonstrated that α- and β-thymosins are involved in immuno-modulation, acting on vascular biology and influencing also cancer pathogenesis [101]. Receptors through which these molecules interact with the cells have not yet been clearly defined, but several studies, over the years, allowed characterizing their physiological functions and their potential role in therapy.

Thymosin α1 (also known as “thymalfasin”; commonly abbreviated Tα1) is an N-terminally acetylated acidic peptide of 28 amino acids with a molecular weight of 3108 Da [93]. It is produced, like FTS, by the RE thymic tissue. Its serum concentration varies by age, but the decrease of Tα1 occurs earlier than that of FTS. Its decline, in fact, starts already around 10 years of age [77].

It is formed by limited proteolysis from its precursor, prothymosin α (ProTα) [99], which is, however expressed in numerous cells [102], at high levels in connection with cell division and, therefore, especially in tumours. The wide expression of ProTα in numerous cells, along with its role as a histone H1-chaperone [95], raises, of course, the question of extrathymic Tα1 secretion, a point that requires further clarification. In fact, it has been found to be expressed, at lower concentrations than in the thymus, in several other lymphoid and non-lymphoid peripheral tissues [103]. Earlier findings of Tα1-immunoreactive peptides in non-thymic cells and tissues and their nuclear localization [104] may have been due to the ubiquitous presence of ProTα.

However, without any doubt, Tα1 is an important immune modulator [105,106], which also exerts actions in the CNS, which seem to be partially due to immunological influences on neuroplasticity [107,108].

By virtue of its immuno-modulatory activities, Tα1 is already used in several therapeutic protocols and clinical trials worldwide, either in monotherapy or in combination with other agents, such as interferon-α (IFN-α) [103,109]. It is mainly applied in the area of infectious diseases, including acute and chronic bacterial infections, fungal and viral infections and also for purposes of vaccine enhancement [103,110,111]. It is also in use against immune deficiencies, caused by senescence or by HIV [103,110], and, both experimentally and clinically, in malignancies [111,112]. Contrary to many other immuno-modulatory drugs, Tα1 has an excellent safety profile, which seems to be related to its anti-inflammatory properties.

With regard to cancer therapies, Tα1 is of particular value, because it attenuates the toxicity of cytostatics and, thus, significantly improves the quality of life in cancer patients [111,113].

In the end, its immunological actions concern both the innate and the adaptive immune systems, by modulating especially activities of macrophages, neutrophils and dendritic cells [114].

Among the β-thymosins of TF5, 20 short peptides have been isolated, which are composed of 40–44 amino acids. However, they do not appear to be thymus-specific but are found in many haemopoietic and non-haemopoietic tissues including bone marrow, spleen and lungs [115]. They all exhibit a cytoplasmic localization but, among them, only the actin monomer-binding species, Tβ4 and Tβ10, are normally expressed in a healthy body [101,116]. In particular, Tβ4, a highly conserved 43 amino acidic peptide, is widely distributed in mammalian tissues and it is involved in numerous processes, such as regulation of cell motility, differentiation, angiogenesis, control of metalloproteinase activity, anti-inflammatory actions, activation of cardiac progenitor cells, acceleration of wound healing, and organization of the cytoskeleton by sequestering G-actin [116]. It is also able to regulate neurogenesis, tangential expansion, tissue growth and hemisphere folding [117]. It is actually regarded as a promising therapeutic agent with healing properties in skin, cornea and cardiac repair [118,119,120].

**Figure 4:**
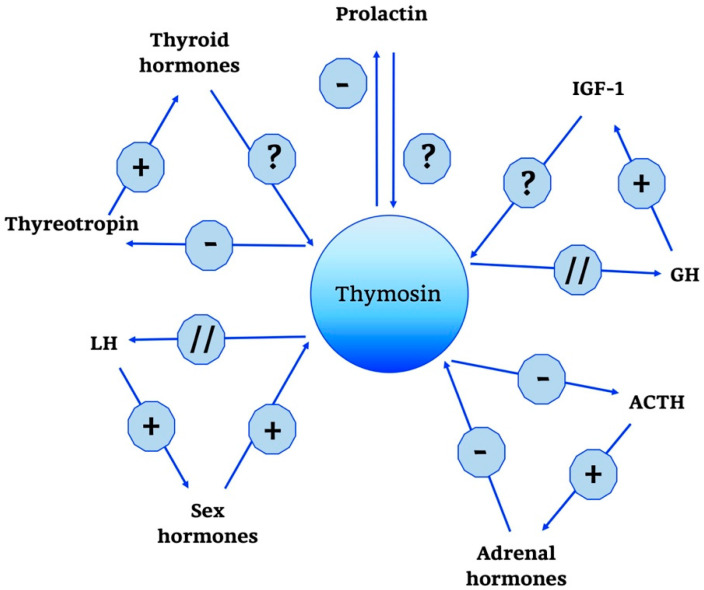
Thymosin neuro-endocrine functions. Thymosin neuro-endocrine functions are summarized in the scheme. +, stimulatory effect; -, inhibitory effect; ?, no data; // no interaction; GH, Growth Hormone; IGF-1, Insulin Growth Factor-1; ACTH, AdenoCorticoTropic Hormone; LH, Luteinizing Hormone.

Thymosins are also part of a wider neuroendocrine network, as shown in Figure 4.

In particular, a predominant role is played by Tα1; some studies have shown that it does not have a direct effect on the adrenal gland, but acts indirectly by modulating hypothalamic activity. In particular, unlike thymulin, it has a predominantly negative role, suppressing the production of the hypothalamic releasing hormones thyrotropin releasing hormone (TRH), corticotropin-releasing hormone (CRH) and somatostatin. Moreover, data confirm that when applied intra-ventricularly, Tα1 decreases plasma ACTH, thyreotropin and prolactin but does not change GH levels [121,122]. In the end, other studies confirm the role of Tα1 in the hypothalamic-pituitary-adrenal axis and also indicate that the interactions between endocrine circuits and Tα1 are differential: while corticosteroids decrease the production of Tα1, estradiol augments the expression of [121,122].

#### 2.2.3. Thymopoietin

Thymopoietin (also known as TP or TMPO) was first described as a 5-kDa, 49-amino-acid polypeptide [123], which was later interpreted as a proteolytic fragment of (a) larger precursor(s) [124]. It is ubiquitously produced, mainly by organs of the immune system, but is also expressed in other cells, especially those showing high rates of proliferation [125]. However, it is the third main product of thymic epithelial cells, but has also homology to the rat lamin-associated polypeptide 2 (LAP2) [126], which has additional functions by directly interacting with B-type lamins in the nuclear lamina and with chromosomal proteins [127], where one of its sub-forms scaffolds histone deacetylase 1 (HDAC1) and is, therefore, involved in epigenetic gene regulation [128]. TP exhibits an early decline in its serum concentration: as described for Tα1, the level of TP also begins to decrease in early life, already around 10 years of age [77].

Importantly, the *Tp/Lap2* hnRNA precursor is alternatively spliced. Proteins resulting thereof have been first described as thymopoietin α (TPα, 75 kDa), thymopoietin β (TPβ, 51 kDa), and thymopoietin γ (TPγ, 39 kDa), with some variability in length according to species [121,125]. Later, seven splice sub-forms deriving from the *Tp/Lap2* gene have been detected, named as TPs α, β, β′, γ, δ, ε, ζ [129], among which the newly discovered forms may be related to other, e.g., LAP2 functions. Several studies on these molecules identified common and unique domains, suggesting that they might have specific functions in relation to distinct subcellular compartments. Specifically, TPs α, β and γ lack classical N-terminal hydrophobic signal sequences that are usually necessary for secretion. Therefore, these molecules may be largely localized intracellularly and exert mainly intracellular functions, which has to be the case for the LAPs anyway [124].

The first data about TP activities have been published in studies carried out to better define its role in neuromuscular transmission. Further investigations have allowed better clarifying the immunological function of these molecules. In particular, TP was found to induce differentiation of prothymocytes to thymocytes. Moreover, it was possible to confirm TP’s ability of enhancing the allogeneic responses of peripheral T cells [124,130].

Functions similar to those by TP have been identified for a synthetic pentapeptide, thymopentin [131], which is composed of residues 32–36 (Arg-Lys-Asp-Val-Tyr) of the first-described 49 amino acid sequence. It obviously contains the decisive binding site for TP targets and, therefore, exerts the same biological activities as the full-length molecule. However, it is rapidly degraded in the blood plasma, with a half-life of about 5–6 min.

Thymopentin has been shown to directly bind to MHC class II molecules on antigen-presenting cells [132]. It has also been reported to stimulate, in murine peritoneal macrophages and spleen lymphocytes, the formation of IL-1α, IL-2, IL-6, IL-10, IFN-γ, nitric oxide, and heat shock proteins (HSP70 and HSP90), These mainly pro-inflammatory actions have been explained by activation of NF-κB pathway, via phosphorylation of Rel A and IF-κB.

**Figure 5:**
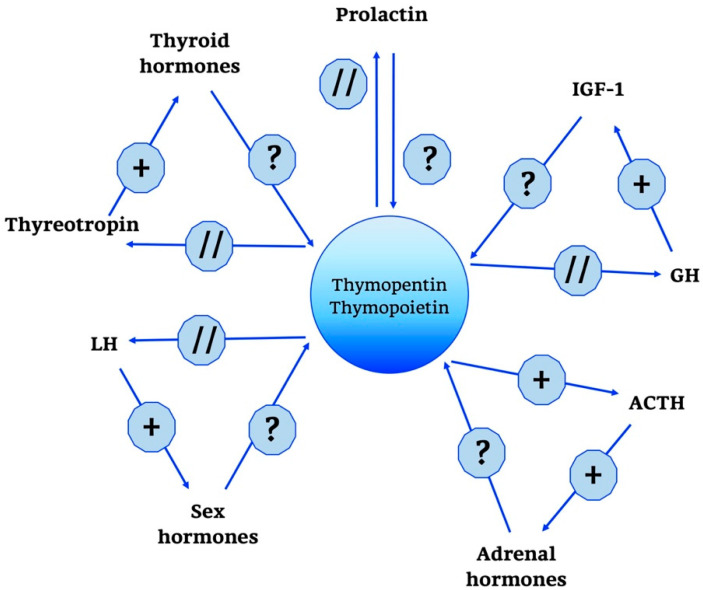
Thymopentin and Thymopoietin neuro-endocrine functions. Thymopentin and Thymopoietin neuro-endocrine functions are summarized in the scheme. +, stimulatory effect; ?, no data; // no interaction; GH, Growth Hormone; IGF-1, Insulin Growth Factor-1; ACTH, AdenoCorticoTropic Hormone; LH, Luteinizing Hormone.

Compared to what has been addressed so far, the neuroendocrine network of TP is rather limited, as shown in Figure 5. The only action of relevance seems to be the one towards the ACTH/glucocorticoid axis and related hormones. In cultivated pituitary cells, in fact, TP and thymopentin increased the levels of ACTH, β-endorphin and β-lipotropin [121,133,134].

#### 2.2.4. Thymic Humoral Factor

Thymic humoral factor (THF), produced by the TG, is recognized as one of the agents that are able to restore humoral and cell-mediated immunity deficits in vivo. Again, the history of this factor had started with a crude peptide fraction [135]. The active ingredient was finally identified as Thymic humoral factor-γ2 (THF-γ2), the only component in tested fractions that turned out to be active in all required in vitro and in vivo bioassays [136]. This molecule is an octapeptide with a molecular weight of 918 Da and the amino acid sequence Leu-Glu-Asp-Gly-Pro-Lys-Phe-Leu that has no homology to the sequences of any other thymic hormone [136].

Overall data confirm that THF-γ2 enhances the proliferative activity in human normal bone marrow and peripheral myeloid and erythroid progenitor cells. In ageing mice, the numbers of mitogen-responsive T cells in thymus and spleen as well as the frequency of cytokine-producing splenic T cells were increased to levels observed in young animals. In the old mice, the efficacy of THF-γ2 in raising T helper cell activity was 400-fold greater than that of thymosin-α1 [137]. The immuno-restorative capacity of THF-γ2 was not only demonstrated in immunosenescent mice, but also in other models and cases of immunodeficiency, such as melphalan-treated mice [138], in human T cell deficiency [139] and bone marrow explants from HIV infected patients [140].

The area in which the activities of THF-γ2 is best understood and in which it is most widely used is immunotherapy in conjunction with chemotherapy for arresting of tumour and metastatic growth [136,141].

To date, the possible role of THF-γ2 in a wider neuroendocrine network has not been investigated.

## 3. Revisiting the Pineal Gland

PG is a circumventricular organ deriving from the embryonic forebrain and it is the major component of the epithalamus together with the habenular nuclei [142,143]. It has an oval shaped structure [38]; its size is anatomically variable and the average weight in human is around 150 mg [144].

The PG is present in almost all vertebrates, with a few exceptions, e.g., an agnathan, *Eptatretus burgeri* (Myxinidae), whereas other agnathans possess this organ [145]. In *Eptatretus*, a diencephalic structure seems to represent a pineal homolog. In the non-vertebrate chordate genus *Branchiostoma*, the lamellar body has been assumed to represent a pineal homolog, a conclusion that has been disputed on the basis of gene expression [146]. An absence of PG has been reported for crocodiles and, in mammals, for dugongs and some Xenarthra such as armadillos, which all possess homologous brain structures [147].

Moreover, MT-secreting brain structures that may be regarded as functional analogs of the PG have been detected in some insects [148]. The PG is considered to be very important for survival of vertebrates [148,149,150]. It has been observed that the PG increases in size in vertebrates from the equator to the poles, which has been discussed in the context of absence of a parietal eye and thermal regulation, though with caution [147], whereas a relationship to the duration of night in winter might be also suggestive. A large body of evidence has shown a clear relationship between the size of PG and pathological conditions; the latter, in fact, alter the cytoarchitecture of the gland [150]. For example, the size of PG is reduced in obesity, in patients with primary insomnia and if there are some changes due to environmental factors [151,152].

After a period of initial growth, the size of the gland remains stable from age 2 to 20 years [153]; thereafter, the weight of PG gradually falls till the second half of mature age and is remains stable in old age [154]. Although the weight does not decrease anymore, the parenchyma changes its morphology; this is frequently related to the amount of calcified deposits that cause alterations and losses in pineal metabolism, in particular, decreases in the production of peptides and indoleamines [155].

### 3.1. Principal Characteristics of Mammalian Pineal Gland

The cytoarchitecture of this gland has been extensively studied from the 20th century to present [38,156,157,158,159].

The anatomical studies have provided more information regarding cell types and innervation of this gland [157]; briefly, the latter are reported below.

In all mammals, many cell types exist: (a) the pinealocytes producing indoleamines; (b) the interstitial cells; (c) microglia, often referred to as perivascular phagocytes, although microglial cells display more functions than that of phagocytosis; (d) the neurons. Additionally, vascular cells and some poorly defined other cells are found in the gland [160].

**Figure 6:**
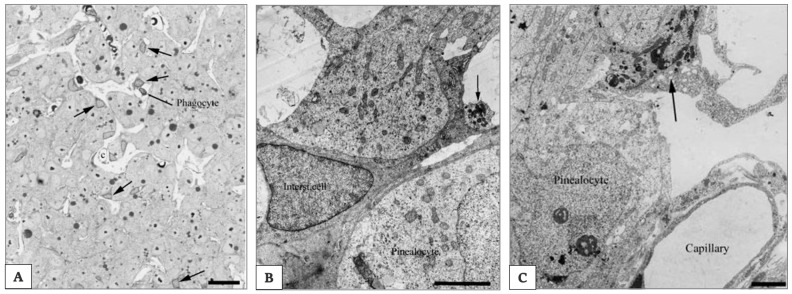
Electron photomicrograph of rat pineal gland. In (**A**), photomicrograph of the rat pineal gland showing ovoid pinealocytes arranged in cords separated by the capillaries and the perivascular spaces. Interstitial cells present a star-like shape and are characterized by their triangular dark nucleus and cytoplasm. In the perivascular space is possible to identify a phagocyte. Arrows indicate the intersitial cells. Scale bar 3.5 μm. Arrows indicate the perivascular phagocyte. In (**B**) electron photomicrograph of the rat pineal gland showing an interstitial cell located between pinealocytes characterized by a more electron-lucent cytoplasm. Scale bar 5 μm. Arrow indicates a club-shaped terminal of a pinealocyte process. In (**C**) electron photomicrograph of the rat pineal gland shows a perivascularly located phagocyte characterized by a high number of dense bodies in the cytoplasm. Scale bar 3.5 μm. Arrows indicate the perivascularly located phagocyte. Pictures are obtained from the work of Møller and Baeres, 2002. Reprinted by permission from Springer Nature Customer Service Centre GmbH: Springer Nature, Cell and Tissue Research, The anatomy and innervation of the mammalian pineal gland, Møller and Baeres, [157].

*Pinealocytes* make up about 95% of the pineal cells in the mature, non-aged organ (Figure 6A) [160]. They show three to five processes emerging from their cell body and showing dense-core granules in club-shaped terminals; these processes known as synaptic ribbons have been identified by electron microscopy (as shown in Figure 6B). The synaptic ribbons have been considered as markers of the pinealocytes and they show dense-core granules in the club-shaped cell terminals. However, in several mammalian species such as rat and guinea pig, these markers are absent or no more detectable. Pinealocytes express the decisive enzymes of MT synthesis, AA-NAT and ASMT [161,162].

*Interstitial cells* are actually regarded as a type of astrocytes [160]. They are smaller than pinealocytes and show a nucleus with a triangular form and a dark cytoplasm (as shown in Figure 6B). They have many longer and slender processes with a higher number of filaments. Most of them are immunoreactive for glial cell fibrillary acid protein (GFAP) [163], which is the main intermediate filament protein in mature astrocytes [164].

*Microglial cells* are mainly present in the perivascular spaces and resemble those in other parts of the central nervous system (as shown in Figure 6C).

The fourth cell type comprises classic neurons and peptidergic neuron-like cells; the first are parasympathetic neurons innervated by a peripheral ganglion [165], while the second ones are positive for neuropeptides such as enkephalin, vasopressin or oxytocin depending on species [157,166,167].

Importantly, not to forget, the mammalian PG is innervated by post-ganglionic fibres of the sympathetic nervous system that originate in the superior cervical ganglion, which receives its day/night information by a neuronal connection from photosensitive melanopsin-containing retinal ganglion cells [168] via retino-hypothalamic tract, SCN, paraventricular nucleus and intermediolateral cell column of the upper thoracic cord [169]. The SCN-controlled rhythm that leads to the mainly nocturnal MT synthesis depends largely on the norepinephrine release by the sympathetic fibres. Additionally, other neuronal transmitters are modulating PG activity, such as neuropeptide Y (NPY), pituitary adenylylcyclase activating peptide (PACAP), vasoactive intestinal polypeptide (VIP), and glutamate [170].

### 3.2. Pineal Ageing

A temporal program about life, ageing and death in the pineal network of the brain has been repeatedly addressed [3,29]. Moreover, PG ageing has been considered responsible for promoting ageing of the body [29]. According to this assumption studies suggested that alterations of pineal metabolism, deteriorations of the circadian system and calcifications that are found in several species contribute to functional losses of the PG, phenomena that aggravate with ageing and are also associated with human pathologies [171,172]. While the focus will here be put on the structural changes in the PG, especially calcification, it should be also noted that decreases in the circadian input from the SCN, either by neurodegeneration in the master clock or by impairments in the neuronal pathway toward the PG, have been discussed as alternate or additional causes of pineal malfunction [173].

Morphological changes in the aged rat PG showed (i) an increase in the thickness of the connective tissue capsule, (ii) an increase of striate muscle fibres that are present in both capsule and parenchyma, (iii) increased numbers of vacuoles, dense bodies and dense vesicles in pinealocytes, (iv) an increase in size of cytoplasmic lipid droplets and presence of interstitial adipose lobules, (v) the presence of myelin-like structures, (vi) an increase in the number and size of calcifications.

The calcifications are called “*corpora arenacea*”, “*brain sand*”, “*acervuli*”, “*psammoma bodies*” or “*pineal concretions*”. In particular, there are two types of calcifications: the polycrystalline berry-shaped complexes and crystalline concretions; these crystals are composed of calcium, magnesium and ammonium phosphates (the first one also as hydroxyl apatite), and calcium carbonate (calcite) [38,174,175]. These calcifications have been sometimes, inappropriately, described as *renal glomerulus* [38]. In young and in old people, they differ in size. In younger patients, the calcifications are globular and localized near the pinealocytes, whereas in older patients, the calcifications are more concentric and associated with glial cells [176]. When age advances, the number of pinealocytes decreases, glial fibres increase, the pattern of calcifications changes.

Where large amounts of pineal parenchyma is present and in younger people, globular calcified deposits can be identified. On the contrary, in cases with large amounts of glial fibres and in older age groups, concentric lamellated calcified deposits are normally found. Correspondingly, this is associated with reduced secretory products of pinealocytes. Thus, the degree of pineal calcification indicates PG malfunction, which is often associated with various neurodegenerative disorders, such as Alzheimer′s disease [177], multiple sclerosis [178] and other pathological conditions [179].

**Figure 7:**
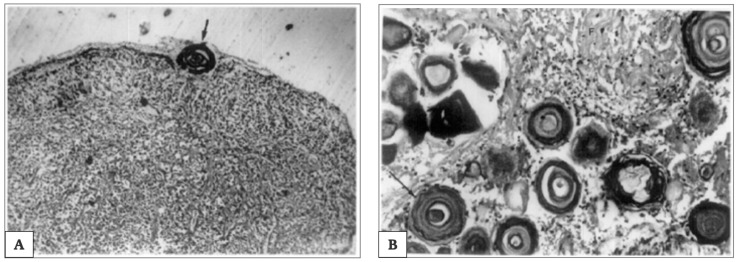
Extrapineal calcifications during pineal aging. Photomicrograph, showing extrapineal calcification in capsule (100×) and concentric lamellar pattern; in (**A**). Thick arrow indicates the lamellar concentric pattern of extrapineal calcification’s development. Photomicrograph showing intrapineal calcification in parenchyma (100×), associated with a concentric lamellar pattern surrounded by glial fibres; in (**B**). Arrow indicate the concentric lamellar pattern. Picture is obtained from the work of Koshy and Vettivel, [176]. The permission to reproduce the material has been obtained from the license holder.

**Figure 8:**
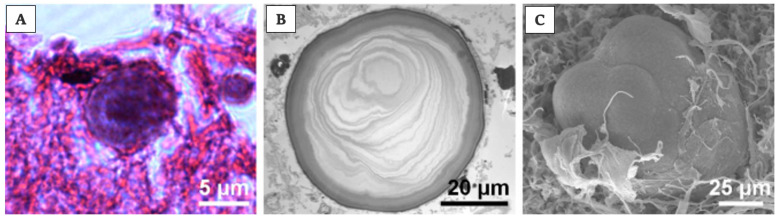
Acervuli’s structure and development in human. Photomicrographs of acervuli in human, showing, in particular, in (**A**) typical concentric ring structure of an acervulus, surrounded by connective tissue; scale bar 5 μm. In (**B**) acervulus with many concentric laminations; scale bar 20 μm. In the end, in (**C**) mulberry-like structure with lobes; scale bar 25 μm. Pictures are obtained from the work of Kim et al., [180]. The permission to reproduce the material has been obtained from the license holder.

Of note is that the “*acervuli*” can show a different morphology, as reported by Kim et al. [180]. Using light and transmission electron microscopy, the authors have shown that these concretions have concentric ring laminations surrounded by connective tissue (as shown in Figure 7A,B) and, using scanning electron microscopy, they visualized Mulberry-like structures with lobes (as shown in Figure 8A–C).

**Figure 9:**
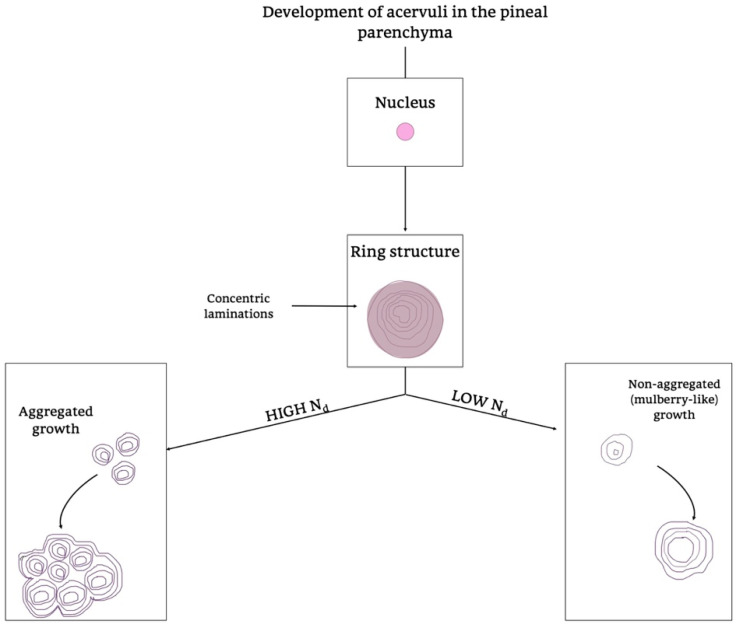
Development of acervuli in the pineal parenchyma. Schematic representation of the formation of acervuli in the PG parenchyma. Two different growth patterns are summarized in the diagram, considering the presence of high or low levels of Nd, a determining factor in directing the different Acervuli’s organization. Nd, Nucleation density.

Moreover, they showed morphology and growth patterns of “*acervuli*” 3-dimensionally by means of synchrotron X-ray imaging. According to these results, authors suggested that the nuclear density (N_d_) plays an important role in the development of their cytoarchitecture [180]. After the formation of ring structures around the accretion core, the acervular morphology can gain two different patterns in relation to N_d_; high N_d_ induces large scale laminae, whereas low N_d_ results in a mulberry-like structure. Thus, high N_d_ promotes the coalition with neighboring “*acervuli*” with same N_d_, as the growth proceeds, and they become aggregates of large size (as shown in Figure 9). These findings are important for interpreting the alterations of concretions under normal and pathological conditions, respectively, in all mammalian species [180].

In conclusion, anatomical changes of pineal cytoarchitecture may be considered either as markers of degradation or as normal stages in the ageing process [181]. A literature review provides conflicting data in this regard; in fact, given the high incidence of calcifications in humans and considering the functions of the gland, Tan et al. have suggested that they should not be considered as a physiological process, but rather a pathological condition associated with ageing [148].

### 3.3. Pineal Products and Their Effects

During the years, most studies conducted on PG’s neuroendocrine activity have focused on its role as the source of circulating MT. However, other studies have hypothesized that some functions of PG, in particular, those linked to ageing, age-related diseases and carcinogenesis, may not be generally mediated by alterations in MT only [182,183]. In this regard, respective studies indicated an important expansion of the endocrine role played by the mammalian PG. This might imply a multiply mediated modulation of the entire neuroendocrine system [184]. Attention was more recently paid to an aspect that had been neglected for years. After the identification of MT as the main indoleamine produced by this gland, in fact, the possible existence of other secretory products with biological activities produced by the PG had rarely been further investigated. Different types of molecules have to be considered in this regard: (i) other indoleamines and their derivatives, (ii) polypeptides with demonstrated hormonal function, and (iii) oligopeptides.

Among the first group, all precursors and various metabolites of MT can be found in the PG, such as serotonin, *N*-acetylserotonin, 5-methoxytryptamine, 5-hydroxytryptophol, 5-methoxytryptophol, and *O*-acetyl-5-methoxytryptophol, an MT analog differing only by replacement of the aliphatic nitrogen by oxygen. Although these compounds have been detected in the PG, they are also formed elsewhere in the CNS and, therefore, their appearance in the blood does not tell much about their site of synthesis and release [185,186].

5-Methoxytryptamine has been occasionally discussed as a second hormone of the PG [187,188]. Several effects of this compound have been summarized [189]. However, evidence for its actions as a hormone in the strict sense has not been fully convincing. Nevertheless, effects of 5-methoxytryptamine in the PG itself have been described [190], a line of experimentation that has not been followed by modern techniques. One of the technical problems in studying this agent concerns it rapid destruction by monoamine oxidase A (MAO A). However, pinealocytes only express monoamine oxidase B (MAO B), which does not oxidize 5-methoxytryptamine [189], a finding that would be compatible with the assumed, but still uncertain role as a hormone.

Some biological/pharmacological effects have been also described for 5-methoxytryptophol [191,192,193] and *O*-acetyl-5-methoxytryptophol [189,194]. The release of 5-methoxytryptophol from the PG has, at least, been demonstrated in trouts using superfusion in culture, but other indoles were likewise released [195]. This compound certainly continues to be a candidate for another pineal hormone, but the spectrum of described actions is still too heterogeneous for providing a coherent picture.

Another compound that derives from methoxyindoles in the PG is a tricyclic molecule, 6-methoxy-1,2,3,4-tetrahydro-β-carboline, also known as pinoline [189]. It is formed from 5-methoxytryptamine under incorporation of a C-atom [196,197]. Again, this compound is also formed in other parts of the CNS, including the retina. Numerous actions have been ascribed to pinoline, mostly in the fields of psychoactive effects [198,199] or anti-oxidative protection [200,201]. Despite the ample documentation of actions of exogenous pinoline and its presence in analysed tissues, its classification as a natural agent is problematic, as it is formed non-enzymatically, also during extraction procedures, leading to uncertainties about physiological rates of synthesis [189].

Another category of pineal constituents are peptides of different lengths. It has been reported that peptide extracts and purified peptides isolated from PG may have anti-gonadotropic, metabolic, and anti-tumour activities [202,203]. Several of these conclusions would require substantiation by further investigation. With regard to anti-gonadotrophic effects, which are especially of importance in seasonal breeders, the PG seems to act by secreting two types of signal molecules, indolic compounds (mainly MT) and pineal polypeptides [204].

Among the pineal polypeptides, arginine vasotocin (AVT) is one of the compounds. It was firstly identified in pineal extracts by Milcu and colleagues in 1963, later synthesized and tested in immature rodents, in which it was shown to modulate many aspects of the sexual physiology of the animals [205,206]. Apart from reproductive biology, this hormone was presumably also responsible for the earlier observed anti-diuretic effects of pineal extracts [207].

For many other polypeptides, the details collected to date are not sufficiently clear. Therefore, many authors still regard them as “unidentified pineal anti-gonadotrophic substances”.

Pineal peptides have been objects of several studies. Their production seems to be partly regulated by MT and, perhaps, other indoles. Some of them seem to be important for modulating the crosstalk between immune, endocrine and nervous systems, acting as autocrine, endocrine, neurocrine, and paracrine mediators. They have been reported to be of significant relevance in ageing and several data have been published on the modulation of cell senescence [208].

They have been also considered as players in more complex systems: (i) the pituitary-gonadal system and (ii) the pituitary-adrenal system. In both cases, studies confirmed the anti-steroid actions of PG constituents that occur after the administration of melatonin-free pineal extracts or purified fractions [209,210,211,212]. Thorough biochemical analyses showed some deviations of pineal peptides from those released by the anterior pituitary [213]. Not all peptides present in a pineal extract are necessarily produced by pinealocytes, since some of them are found in peptidergic nerve fibres that contribute to the control of pineal activity [214].

While pineal peptides were a hot topic in the 1970s and early 1980s, this field was more and more abandoned in later years, with the exception of the work by Khavinson and colleagues, who focussed on a pineal tetrapeptide (Ala-Glu-Asp-Gly), which was named epitalon [215,216].

Despite numerous publications on this issue, various details of the actions of peptides remain to be clarified, in particular those concerning the sites of action in a hierarchical endocrine axis. Several effects may be primarily exerted in the brain, but multiple actions at different positions in the hierarchy cannot be *per se* excluded. With regard to the complexity of the networks, the systemic relevance of the peptides remains to be elucidated.

While the role of MT as a protective compound with particular value in preventing age-related pathologies and a potential of contributing to healthy ageing has been multiply documented and reviewed [217], similar effects of pineal peptides have been claimed, but have not yet received the same attention. With regard to demographic developments, the complete arsenal of anti-ageing compounds produced in the body would be of high value. Considering the progressive shift in lifespan prolongation and life expectancy, as well as the resulting increase in the number of patients affected by neurodegenerative diseases such as Alzheimer′s, Pick’s, Parkinson’s and Huntington’s diseases, vascular dementia, ischemic encephalopathy cardiovascular diseases and other angiopathies, the possibility of identifying molecules capable of inducing rejuvenation of nervous, vascular and endocrine tissues is a perspective that has attracted the attention of many investigators.

To date, however, the activities of the endogenous short peptides produced by the PG have not been profoundly analysed. Some studies suggested that they could offer new hopes for the retardation of ageing and treatment of the age-related progressively invaliding diseases [216,218,219,220,221,222,223].

For the future, it will be necessary to thoroughly identify their molecular mechanisms of action, to discriminate multiple pathways and to follow their catabolism, bioavailability and pharmacokinetics. Research of this type would complete our understanding of pineal activity and its systemic role.

#### TRH

With the years the deepening of studies around the PG activity and MT production allowed to understand that the roles played by PG are multiple. In particular, apart from MT and the other, identified or unidentified, peptides reported above, PG also contains the TRH [224,225,226,227] and, additionally, iodothyronine 5′-deiodinase (type-II thyroxine 5′-deiodinase) [228].

Therefore, the PG may modulate thyroid activity in a dual way. Interestingly, the bovine PG was reported to also produce TRH inhibitory factor (TRH-IF), which additionally inhibited prolactin release [229]. By producing and releasing TRH, the PG not only becomes part of another neuroendocrine axis, the PG-thyroid axis, but exerts, via TRH, additional effects on thymus. In athmymic nude mice, TRH restored antibody production [224]. This hormone was also reported to antagonize thymic involution, perhaps by modulating steroid- or stress-related signals [224,225]. In this way, PG could partially restore the immune system activity not only through MT secretion, but also independently through TRH release [226].

Collectively, data on all pineal products, as far as they are really secreted, may serve a wide range of different biological roles, which includes the support of the immune system, geroprotective actions that comprise neuroprotective, anti-atherosclerotic and cancer-preventive activities.

Therefore, MT and other pineal secretory products might be a promising remedy to reverse the age-related endocrine dysfunctions of primates [230,231].

## 4. Melatonin

Melatonin (MT), N-acetyl-5-methoxytryptamine, is a ubiquitous and widely distributed indoleamine that can be found in almost all living organisms [232]. It was firstly isolated in 1958, by Lerner and colleagues, as a molecule characterized by its ability to aggregate melanin granules within the melanocytes; today many researches have shown that MT has many different functions.

It is produced from tryptophan, an essential amino acid, and it could be defined as a messenger in any compartment of the organism. Moreover, it could be considered as an ancient chemical messenger, in particular considering its persistence among different species. Despite this, however, it is still widely studied [233].

Of noteworthy interest is understanding how melatonin interacts with cells and their components. Besides being produced in peripheral tissues and acting as an autocrine and paracrine signal, MT is centrally synthetized by PG with a light-dark regulation. Over the years, studies confirmed that, once released in the bloodstream, MT could work indirectly, through specific MT-membrane-receptor, MT1 and MT2, responsible for its ‘‘chronobiotic’’ effects [233]. Moreover, as for its amphiphilicity, MT is able to cross the cells, organelles, and nuclear membranes and directly interacts with nuclear receptor, and also with intracellular molecules in a non–receptor-mediated actions.

Above all, the presence of MT and its synthesis in organs other than PG is well documented; specifically, TG has been repeatedly confirmed as one of the main MT-producer in human [23,29,67,170]. Details about melatonin produced from TG and PG have been summarized in the following paragraphs.

### 4.1. Thymic Melatonin

The presence of MT and its synthesis in the TG has been repeatedly reported since the end of the last century, in mice, rats, palm squirrels and humans [23,67,234,235,236]. Some studies demonstrated a relatively large amount of MT in TG homogenates, exceeding that present in serum [67], whereas considerably lower concentrations than in the PG were measured [23].

The biosynthetic pathway of MT involves four intracellular steps [237,238] catalyzed by tryptophan hydroxylase (TPH), aromatic amino acid decarboxylase (AADC), aralkylamine-*N*-acetyltransferase (AA-NAT) and *N*-acetylserotonin-*O*-methyltransferase (ASMT; formerly known as hydroxyindole-*O*-methyltransferase, HIOMT). Intra-thymic synthesis of MT has been demonstrated in several of the above-mentioned papers by the expression of *Aa-nat* and *Asmt* mRNAs and presence of the corresponding enzymes. This conclusion was further corroborated by *Aa-nat* expression in mouse strains that are pineally melatonin-deficient, e.g., the C57BL/6 sub-strain [236]. Moreover, it has been shown that pinealectomized rats have an increased thymic MT amount [234], not only confirming the endogenous synthesis, but indicating that TG gains importance as a site of synthesis when pineal synthesis is absent.

The roles of MT in the mammalian body are complex. In this group of animals, circulating MT is mostly of pineal origin, but it is important to keep in mind that much higher amounts are released by the PG via the pineal recess to the third ventricle of the brain, where the circadian master clock, suprachiasmatic nucleus (SCN), is directly accessible [239], a route that has been recently considered to be much more important to the SCN than the blood route [240,241]. The contribution of extrapineal sites of MT formation to plasma melatonin is usually believed to be minor.

With regard to the almost ubiquitous MT formation in the body, this is a remarkable point. Some contributions by lymphoid tissues and circulating or migrating leukocytes can be taken as proved and are in line with MT levels in pineally deficient mice [236]. However, the release of MT from extrapineal sources can be conditional, such as in the case of post-prandial release of gastro-intestinal MT. An extreme example is the effect of tryptophan on circulating MT in humans. Tryptophan injection caused rapid increases in MT levels about 3 times higher than the normal circadian maximum [242]. These augmentations were ascribed to a release by the gastro-intestinal tract (GIT), which contains the highest amounts of MT in the body. However, one might suspect that other extrapineal sources may similarly respond, though in proportion to their size. Moreover, the important immunological role of the GIT and its participation in a neuroendocrine network should be remembered [243]. In fact, a stimulation of MT synthesis has been also reported for the hamster TG, and spleen, too [244], surprisingly and in contrast to the pinealectomized rats [182] by MT administration. At least, this shows again that thymic MT synthesis is modifiable.

In addition to the MT synthesizing enzymes, the MT receptors MT_1_ and MT_2_ have been demonstrated in the TG of various species [234,244,245,246,247,248]. MT receptor expression was also studied in dependence of age. In human children, Cruz-Chamorro et al. showed decreases in both receptors, at mRNA as well as protein levels [247]. In rats of older age, MT receptors decreased in many peripheral target tissues, but interestingly not in the TG, in which increases of both receptors were observed at mRNA level, and maintenance of expression at the protein level [246].

In addition to the changes in receptor expression, decreases in AA-NAT levels were observed from newborns to children of several months (less than 1 year) and older, whereas ASMT expression increased especially above 1 year [247]. Thymic MT decreased especially from newborns to children of several months and was thereafter maintained. As the patterns do not precisely match, one might think about the way by which AA-NAT and ASMT complement each other. The possibility of differences in rate limitation, as known from the normal pineal MT peak or—partial—inversion of the enzymatic steps, as demonstrated in other species [249], may be too speculative without direct investigation. This should also apply to the discussed correlation of AA-NAT and MT receptor dynamics [247], which would have to be analysed by considering the roles of circulating MT and autocrine/paracrine signalling.

Moreover, MT-membrane receptors were suggested to influence cytokine production and cell proliferation in the immune system [245,246,250,251]. In a general sense, this is certainly so and in agreement with countless influences of MT on cytokines in a pathological context and the modulation of M1 vs. M2 macrophage polarization. To what extent this applies to the TG would require further investigation.

**Figure 10:**
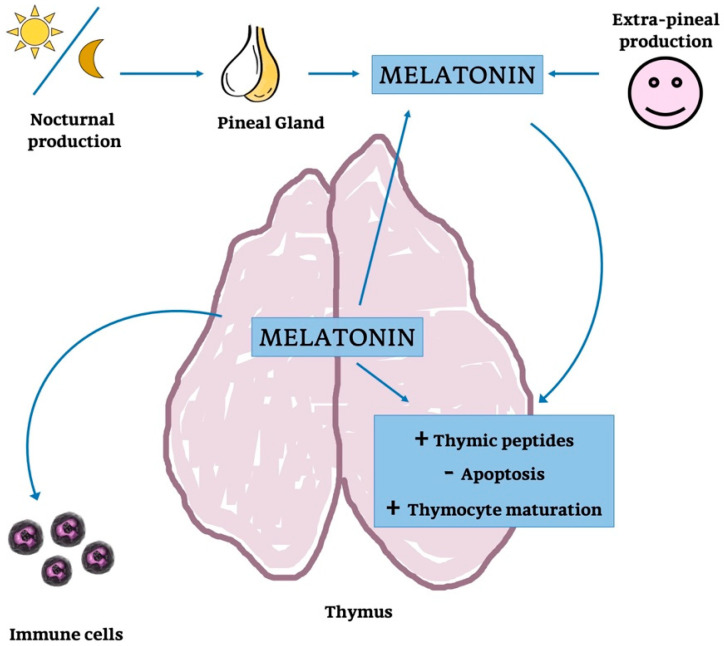
Melatonin’s action on thymus function and parenchyma. Schematic representation of the possible action of MT on thymic parenchyma and function: MT is produced by PG but also provided by systemic production. MT released into the blood stream could modulate thymocytes maturation, cytokine concentration and it could also might regulate thymic peptides.

In the end, the endogenous thymic MT synthesis seem to be regulated by circulating levels of MT (Figure 10); this would suggest that endogenous and circulating levels of MT are functionally strongly associated, as concluded for other organs [252,253,254].

### 4.2. Pineal Melatonin

Of all the functions of PG, its role as the main site of MT secretion has been the by far prevailing aspect over the years.

Many studies have addressed the multiple actions of this molecule. This extends beyond normal physiology to numerous aspects of health including counteraction and prevention of pathologies up to healthy ageing. In particular, the therapeutic indications for which it may be of fundamental importance are constantly increasing. With regard to its extraordinary pleiotropy, it is peerless among other organs and systems in the human body [255,256].

MT is a regulator molecule that influences different functions in numerous organs and cell types in the whole organism, acting, via MT_1_ and MT_2_ receptors, through several, partially parallel, but also diverging signalling pathways [257]. Of course, modulation of metabolism has to take place at different levels, from genetic and epigenetic regulation of transcription, post-transcriptional processes, translational control, post-translational modification, activation/inactivation of enzymes and other proteins via second messengers to influences by secondary signalling mediated by up-regulating other factors. All this is found in the spectrum of MT’s actions. Two particular aspects that exceed the classic view of melatonin’s actions shall be emphasized: (i) The conditional up-regulation of SIRT1 by MT leads to phenomena of an extended, secondary signalling by this sirtuin, which comprises anti-inflammatory, anti-oxidant and circadian effects [258,259]. (ii) The discovery of MT receptors in mitochondria, in addition to mitochondrial MT synthesis, in the so-called mitocrine role of MT, has opened additional metabolic and protective aspects that are presumably applicable to all MT-synthesizing cells [260].

Our view of MT’s role has considerably changed, from the movement of melanosomes in the skin lightening response of lower vertebrates, followed by an immense body of knowledge on circadian and reproductive cycles, to a more comprehensive understanding as a systemic regulator, with actions in the fields of neuroscience/neurodegeneration, endocrinology, immunology, nutrition, anti-oxidative protection, pathogen control and gerontology. Its actions also extend to the control of, e.g., ion channels, neurotransmitter release, stem cell differentiation [261,262,263] mitochondrial functions including maintenance of electron flux [264,265,266,267], and the complex of mitophagy, autophagy and prevention of apoptosis/pyroptosis [268,269,270,271,272].

Among these fields of action, anti-oxidative and anti-inflammatory protections have received highest attention. Again, MT’s effects cover a broad range pf mechanisms, from scavenging of free radicals to modulation of the expression of genes relevant to redox metabolism, multiple forms of radical avoidance, safeguarding of mitochondrial electron flux and mitochondrial [273,274,275,276,277,278].

**Figure 11:**
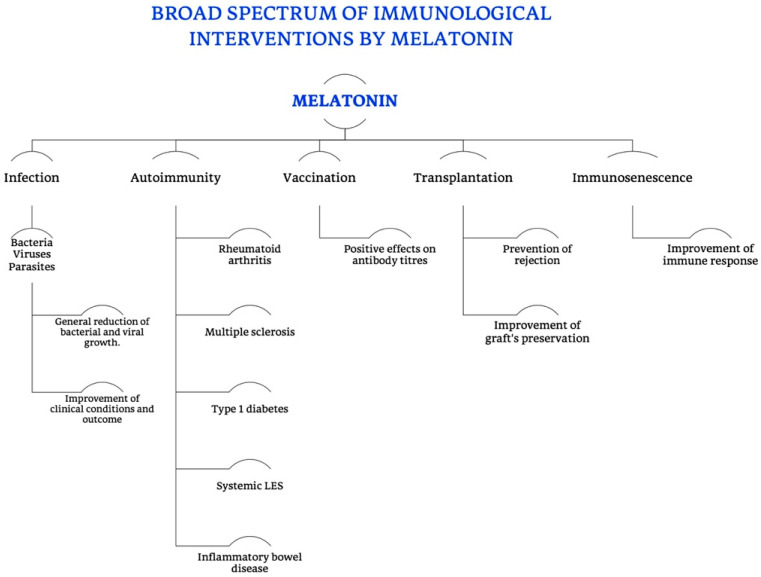
Broad spectrum of immunological interventions exerted by melatonin. Summarizing scheme of all the different functions of MT, in particular in the immune and immunomodulatory field, specifying its different roles in preventing symptoms and complications of infections (bacterial, viral and parasitic), in modulating the progression and outcome of autoimmune diseases, cross immunity and rejection in transplantation, as well as the immunosenescence, finally intervening in the humoral immunity.

MT’s pleiotropy, recognized by many authors, is mainly due to two specific characteristics of this molecule (as shown in Figure 11).

On the one hand, its production extends to different sites, not only PG, which means that other organs—including TG–possess an amount of endogenous MT, which can in some tissues exceed that of the PG [67]. On the other hand, it is able to act both through receptor-mediated and direct radical scavenging. In the first case, the almost ubiquitous presence of its receptors within the vertebrate body is the basis of its cell biologically regulated pleiotropy. The classic membrane-associated, G protein-coupled MT receptors are in mammals represented by MT_1_ and MT_2_. These are not only responsible for the ‘‘chronobiotic’’ effects of MT [243], but also for the majority of its actions. Other MT binding sites have been also identified, even if they are still poorly understood or almost unknown [279]: (i) a previously assumed “MT_3_” receptor has turned out to be the enzyme NRH quinone oxidoreductase 2 (NQO2) [280], but the binding site is unspecific for MT and no convincing signal transduction mechanism is known, what should be expected from a true receptor; (ii) a binding site in calmodulin (CaM) [281] is still in the debate, since the strongly diverging affinities of some studies may be explained by increased MT binding to Ca^2+^/CaM-activated enzymes; (iii) other, poorly defined binding sites are listed elsewhere [282]. The judgment on transcription factors of the retinoic acid receptor family, especially retinoid orphan receptors (RORs), such as RORα, but also other splice variants and homologs, which had been regarded for several years as nuclear MT receptors, has meanwhile profoundly changed. Although this function had been controversial from the beginning, despite lack of confirmation by other groups and the 1997 retraction of main results in the key publication [283], numerous authors continued to regard these molecules as nuclear MT receptors, unfortunately also in many, otherwise valuable, immunological papers. With regard to the large body of publications, this opinion was also divulgated by numerous reviews. After RORα was definitely shown to not bind MT [284], the role as a nuclear receptor has been abandoned and appears unlikely for its homologs. Therefore, the respective data have to be newly interpreted. One possibility may consist in the SIRT1-dependent effects on RORα binding to its response elements [284].

The majority of earlier studies on pineal-derived MT had focussed on its role as a regulator of circadian and seasonal rhythms, especially as MT is mainly synthesized and released into blood at night. Therefore, the cyclicity of its synthesis and secretion had to be first elucidated [67,230,285].

Besides these considerations, research on MT has become increasingly directed towards clinical applicability [231]. In particular, MT’s protective function has been shown under numerous clinical conditions and corresponding animal models, such as ischemia/reperfusion injury in brain, heart, lung, kidney and GIT/liver [286,287,288,289], inflammatory bowel diseases [290,291], toxicity of anti-cancer treatments such as chemotherapy [292] and radiotherapy [293], and many other diseases. It has also been found to be suitable as an adjuvant in radiotherapy [231]. Acting as a potent anti-oxidant and immuno-modulator MT is able to scavenge free radicals, to reduce free radical formation, to boost DNA repair mechanisms [294,295], and to modulate the immune cells responses after ionizing radiation.

Due to these anti-oxidant and anti-inflammatory properties, it serves as a highly effective and well tolerated radioprotective agent in normal tissues [67,231,296], but surprisingly acts concomitantly as a radiosensitizer in tumours, presumably in relation to other anti-tumour activities [231].

**Figure 12:**
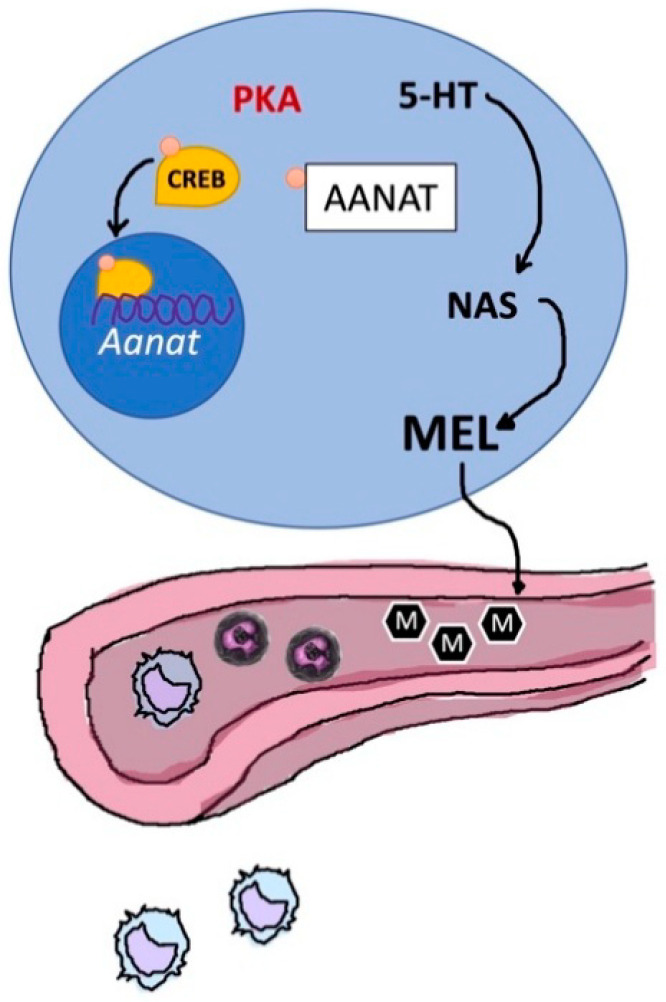
The immune-pineal axis. Schematic representation of the relationship between thymic (in blue) production of melatonin and its secretion into the bloodstream; Aanat, aralkylamine-N-acetyltransferase; 5-HT, 5-hydroxytryptamine; ML, melatonin; NAS, N-acetylserotonin; PKA, protein kinases A; CREB, cAMP response element-binding protein.

Other progress was made by studies which confirmed that ageing and age-related diseases are characterized by reductions in MT secretion and concentration, causing changes in the immune system, deteriorating the circadian multi-oscillator system and its numerous functions. Moreover, data confirm that the rhythmic production of MT also corresponds to rhythmic actions at the peripheral level. In particular, the daily mobilization and proliferation of bone marrow stem cells, for examples, indicates a strict relation between MT circadian production and its impact on the immune system [231]. However, these authors also suggested that up-regulation of NF-κB under inflammatory conditions inhibits AA-NAT expression in both pinealocytes and immune cells, thereby interrupting the immune-pineal axis, which allows the pineal to recover, when the inflammatory input decreases, effects they may lead to a shuttle between active phases of PG and immune cells [231], (Figure 12).

## 5. Concluding Remarks

Mutual influences and bidirectional networks among the nervous, endocrine and immune systems are mediated by hormones, cyto-/chemokines and their binding sites, contributing, in turn, to body homeostasis [23,297,298]. Many interactions are mediated by the highly regulated release of several hormones and peptides into the vessels, which have direct effects on the immune system. In particular, many studies have reported interactions between PG, TG and other parts of the immune system owing to the widespread expression of receptors that detect the respective humoral signals [299].

As stated above, the impairment of TG and PG functions, mainly related to neuroendocrine and immune systems, is associated with ageing, and unfortunately, the physiological deterioration is a precursor to pathologies. Moreover, the alterations of the TG-PG axis during ageing are already indicative of a close relationship between these two glands. In fact, the PG, certainly via MT, but perhaps also via additional factors such as TSH and other peptides, influences humoral and cellular immunity, immune cell proliferation and immune mediator production. The TG is central to many immunological functions, which are mediated by peptides. Moreover, these glands play an important role as a functional unit because they constitute a bidirectional system in which PG acts on TG and *vice versa*. Thus, they have a mutual complementarity in the maintenance of a normal immune and endocrine status, which becomes especially evident in ageing [24].

According to our current knowledge, the mutual functional relationship of the TG-PG axis during the phases of life and, particularly, in ageing would benefit from and be worth of increasing research efforts. To date, the literature is still somewhat scanty and does not easily reveal the entire complexity of their relationships, in part because many findings are relatively old and often published in languages rarely used in science.

It has been the aim of this article to explain and underline the role of the TG-PG axis. Some of the most important aspects, known until now, are summarized here;

MT and TRH were shown to modulate thymic function, to block thymic involution, and to restore immune competence in nude (athymic) mice [225,226]. MT may be involved in thymus regulation by modulating TRH secretion via its receptors present in the hypothalamic areas of the brain [225]. Effects via CRH and glucocorticoids are less likely, since MT was found to not change glucocorticoid levels [299], whereas glucocorticoids were shown to interfere with MT secretion [300]. Nevertheless, MT can antagonize glucocorticoid effects in the immune system [301]. Moreover, it has been suggested that a pineal graft into the thymus acts on its host via MT or via other peptides, which are not well defined, but may be spreading in the thymus [226].

Paltsev et al. suggested that TG and PG have different molecular mechanisms of involution, even if they showed similar alterations in their cytoarchitecture and/or in key proteins related to ageing. This network is due to a relationship between neuroendocrine and immune systems; several factors are released into the vessels with direct actions, or by regulating the secretion of peripheral endocrine glands, which have immune modulating effects, too [23].

Linkova et al. reported an influence of thymic peptides on PG and pineal peptides on TG. Importantly, removal of the PG induces decreases in thymus weight, the number of thymic cells and TG secretory functions [31]. The blockage of PG, induced by pharmacological treatments, also resulted in a decrease of the thymic hormone, thymulin, in the blood [302]. Moreover, thymectomy caused changes in biorhythms for several immune indicators of glucocorticoid synthesis by the adrenal cortex [303].

The bidirectional communication between TG and PG is also due to the release of some cytokines that are secreted by these glands. One of them is tumour necrosis factor-α (TNF-α) which is produced, apart from other immune cells, in the thymic medulla [304]. According to Giroir et al., the biosynthesis of TNF-α in the normal thymus implies a role for this protein in the development and regulation of the immune response [304]. Otherwise, it is known as a major pro-inflammatory factor. Therefore, it is surprising that TNF-α can be considered as a key factor in the pineal-immune axis [305]. Considering this, Markus et al., suggested that this protein plays an important role in the shuttle between endocrine and paracrine MT sources in human acute inflammatory model and in healthy condition [305]. These authors proposed that (i) TNF-α suppresses MT synthesis in rat PG by down-regulating *Aa-nat* expression [306] and that an inverse correlation between this cytokine and the nocturnal MT production exists in the human; (ii) not only MT but also the PG itself plays a role in the modulation of innate immune responses. Under inflammatory conditions (e.g., mastitis), MT production was reported to be shifted from the PG to phagocytes [306]. The reduction of pineal MT may, therefore, be a mechanism allowing an efficient inflammatory response, whereas the role of increased MT from phygocytes may be of local and, perhaps, temporally restricted importance. In fact, the relationships between MT and cytokine producing cells, whether leukocytes or others (cf. SASP), is much more complex and can be considerably different under inflammatory and non-inflammatory conditions. For instance, MT stimulates, under healthy conditions, T-lymphocytes, leading to a nocturnal increase of interleukin-2. Numerous other effects of MT on cytokines have been summarized elsewhere [307].

**Figure 13:**
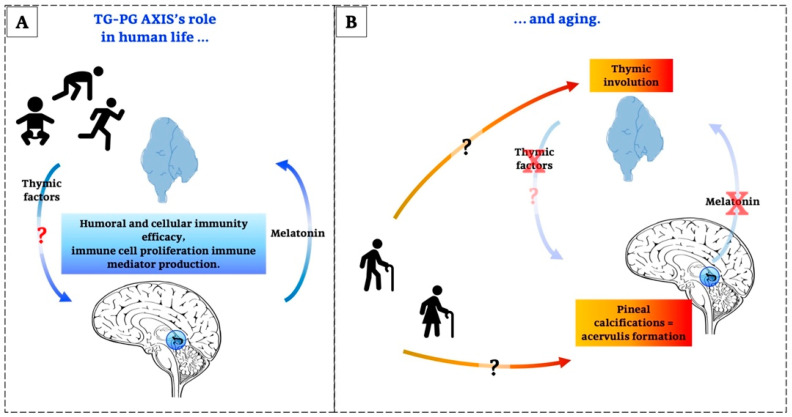
The role of TG-PG axis in human life and ageing. Graphic summarizes in (**A**), the schematic representation of the physiological relationship between thymic and pineal function and activity; in (**B**), the alterations due to the ageing of both the glands and the possible effect of their involution on human health.

Figure 13 summarizes the possible network of TG-PG axis in human life and ageing.

The histological and functional losses of both the PG and TG are salient features of ageing. The message of this article is that they seem to be interrelated. While the deterioration of the PG results in the lack of stimulatory and protective signals to the TG, whereas the thymic involution strongly contributes to immunosenescence and favours inflammatory processes that affect the PG and numerous other organs, too. As far as can be judged on the basis of published data, one may speculate that a solution to maintain the functional integrity of the PG would be to reduce or retard the formation of concretions or to reverse it. Although a respective approved strategy is still missing, this would be worth every effort for preserving the many other organs which are functionally linked to this gland including the TG.

In conclusion, we hope that this review which also recapitulates the functions of the TG-PG axis may help to better understand the role of this system, which deserves more future attention and can open new perspectives in health maintenance and ageing. Moreover, we hope that the information reporter should help to focus the scientific attention on all the products of TG and PG in order to deepen what is known about the role of peptides other than melatonin, both in health and in illness, in young and, even more in old people.

## Abbreviations

PG	Pineal Gland
TG	Thymus Gland
TG-PG axis	Thymus-Pineal Axis
ROS	Reactive Oxygen Species
NAD^+^	Nicotinamide Adenine Dinucleotide
SIRT1	Directory Of Open Access Journals
MT	Melatonin
miRNAs	MicroRNAs
circRNAs	miRNA-Sponging Circular RNAs
DBP	D-Box-Binding Protein
ETP cells	Early T Lineage Progenitor Cells
LIF	Leukemia Inhibitory Factor
OSM	Oncostatin M
GnRH	Gonadotropin Releasing Hormone
LH	Luteinizing Hormone
FSH	Follicle Stimulating Hormone
GH	Growth Hormone
IGF-1	Insulin-Like Growth Factor-1
SASP	Senescence-Associated Phenotype
FTS	Facteur Thymique Sérique - Thymulin
RE	Reticulo-Epithelial Tissue
ACTH	Adrenocorticotropic Hormone
TSH	Thyroid-Stimulating Hormone
sEAE	Severe Experimental Autoimmune Encephalomyelitis
TF3	Thymosin Fraction-3
TF5	Thymosin Fraction-5
Tβ4	Thymosin Β4
Tβ10	Thymosin Β10
Tα1	Thymalfasin - Thymosin Α1
ProTα	Prothymosin Α
INF-α	Interferon-Α
TRH	Thyrotropin Releasing Hormone
CRH	Corticotropin-Releasing Hormone
TP (TMPO)	Thymopoietin
LAP2	Lamin-Associated Polypeptide 2
HDAC1	Histone Deacetylase 1
TPα	Thymopoietin α
TPβ	Thymopoietin β
TPγ	Thymopoietin γ
HSP70	Heat Shock Protein 70
HSP90	Heat Shock Protein 90
THF	Thymic Humoral Factor
THF-γ2	Thymic Humoral Factor-Γ2
GFAP	Glial Cell Fibrillary Acid Protein
NPY	Neuropeptide Y
PACAP	Pituitary Adenylylcyclase Activating Peptide
VIP	Vasoactive Intestinal Polypeptide
N_d_	Nuclear Density
MAO A	Monoamine Oxidase A
MAO B	Monoamine Oxidase B
AVT	Arginine Vasotocin
TRH-IF	TRH Inhibitory Factor
TPH	Tryptophan Hydroxylase
AADC	Aromatic Amino Acid Decarboxylase
AA-NAT	Aralkylamine-*N*-Acetyltransferase
THF	Thymic Humoral Factor
ASMT (HIOMT)	*N*-Acetylserotonin-*O*-Methyltransferase (Hydroxyindole-*O*-Methyltransferase)
SCN	Suprachiasmatic Nucleus
GIT	Gastro-Intestinal Tract
NQO2	NRH Quinone Oxidoreductase 2
CaM	Calmodulin
RORs	Retinoid Orphan Receptors
5-HT	5-hydroxytryptamine
NAS	N-acetylserotonin
PKA	Protein Kinases A
CREB	cAMP Response Element-Binding Protein
